# Nitazoxanide in Acute Rotavirus Diarrhea: A Randomized Control Trial from a Developing Country

**DOI:** 10.1155/2017/7942515

**Published:** 2017-02-26

**Authors:** Samarendra Mahapatro, Nijwm Mahilary, Amit Kumar Satapathy, Rashmi Ranjan Das

**Affiliations:** ^1^Department of Pediatrics, All India Institute of Medical Sciences, Bhubaneswar, India; ^2^Department of Pediatrics, Hi-Tech Medical College and Hospital, Bhubaneswar, India

## Abstract

*Background*. Acute diarrhea is one of the leading causes of childhood mortality, with rotavirus being an important pathogen. Nitazoxanide, an antiparasitic agent, has been shown to inhibit rotavirus.* Objective*. This double-blind, randomized trial was designed to study the role of nitazoxanide in acute rotavirus diarrhea.* Methods*. Of 174 children (12 months to 5 years) with acute diarrhea, 50 rotavirus positive cases were randomized. The intervention group received syrup nitazoxanide twice daily (100 mg in 12–47 months, 200 mg in ≥4 yr) for 3 days along with standard treatment of diarrhea. Duration of diarrhea was the primary outcome measure.* Results*. The median duration (hrs) of diarrhea (54 versus 80; 95% CI: –26 [–13.2 to –38.8]) and hospitalization (68 versus 90; 95% CI: –22 [–12.98 to –31.02]) was significantly shorter in the nitazoxanide group. No significant difference was seen in the median duration (hrs) of fever or vomiting or the proportion of children requiring parenteral rehydration. There was no report of any adverse events.* Conclusions*. Oral nitazoxanide is effective and safe in the management of acute rotavirus diarrhea in Indian children (CTRI REF/2016/10/012507).

## 1. Introduction 

Diarrheal illness is one of the six leading causes of childhood mortality. In developing countries among children under five, it causes 15% of the 10.5 million deaths annually [1]. In India, it constitutes 13% of the common illness in children under five [2]. The global burden of rotavirus diarrhea, predominantly in developing countries, is estimated to be more than 100 million episodes, over 20 million out-patient visits and more than 6,00,000 deaths (range 4,54,000 to 7,05,000) [3]. A multicentric study by the Indian Rotavirus Strain Surveillance Network reported the detection of rotavirus in stools of 39% children aged <5 years [4]. The disease is more severe in infants aged 3 to 24 months. Due to the high incidence of rotavirus infection in both developing and the developed countries, the development of vaccines took shape in the early 1980s. Data on rotavirus vaccine impact in developing countries including India are sparse due to limited use of rotavirus vaccines in this region [5]. Poor access to medical care, malnutrition, and illiteracy are the main factors leading to the higher disease burden in these regions.

The rotavirus diarrhea is treated like any other diarrhea in childhood with oral rehydration therapy (ORT) and zinc [6]. ORT aims to prevent or reverse dehydration and has no effect either on the duration or on the stool output. Zinc is also not universally effective and has been used mainly in developing country settings [7]. For this reason, various other modalities (e.g., probiotics, diosmectite) have been tried with some benefits [8, 9].

Nitazoxanide is an oral synthetic broad spectrum antiparasitic agent that acts by interfering with the pyruvate ferredoxin oxidoreductase (PFOR) enzyme-dependent electron transfer reaction, which is essential to anaerobic energy metabolism [10]. It was approved for pediatric use by the US Food and Drug Administration (FDA) in year 2002, and the first clinical trial on rotavirus diarrhea was conducted in year 2006 [11]. The trial was based on the fact that an active metabolite of nitazoxanide (tizoxanide) showed a cytoprotective effect in in vitro rotavirus infected cells. After that two more trials (each one in children and adult) have been published, all showing beneficial effect [12, 13]. This drug can be an effective low cost treatment to control rotavirus diarrhea till the vaccination is universal in developing countries. There is no published study from developing countries including India specifically assessing the therapeutic efficacy and safety of nitazoxanide in acute rotavirus diarrhea. Hence, the present study aimed at evaluating nitazoxanide as a possible therapeutic intervention in acute rotavirus diarrhea in Indian children.

## 2. Materials and Methods

This double-blind, randomized controlled trial was conducted in the Department of Pediatrics of Hi-Tech Medical College and Hospital, Bhubaneswar, India from April 2014 to August 2014. Children with acute onset diarrhea of <48 hrs duration, some to severe dehydration, and aged between 12 months to 5 years were eligible for inclusion. Diarrhea was defined as passage of ≥3 unformed or loose stools in the last 24 hours [6]. Children with dysentery, severe malnutrition (weight for height < 3 SD of WHO growth-chart), coexisting systemic illnesses, and chronic diseases were excluded from the study.

### 2.1. Methodology

Children with acute diarrhea and positive stool rotavirus antigen test (SD Bioline Rota kit test followed by RT-PCR) were included. They were randomized to receive either nitazoxanide (intervention group) or no nitazoxanide (control group) through a computer generated admission list. Sequence was generated by a person not directly involved in execution of the study. Allocation concealment was done using serially numbered opaque sealed envelopes. The study was approved by the institute ethics committee. Informed written consent was obtained from the parents/legal guardians prior to enrolment in the study.

Prior to admission into the study, history, physical examination, nutritional status, hydration status (as per WHO guidelines), fever, oral acceptance, and stool characteristics were recorded on a predesigned pro forma. Case management was done as per the WHO acute diarrhea guideline [6]. After clinical stabilization and hydration maintenance, children were randomized into two groups: intervention group received syrup nitazoxanide twice daily (100 mg in 12–47 months; 200 mg in ≥4 yrs) and control group received a similar product, both being administered twice daily for 3 days. Both intervention and control had similar color and taste. The children were discharged after improvement in their clinical condition and were followed till day 7. During hospitalization, they were monitored for frequency and consistency of stools and time since last loose stool (every six hours/24 hrs). They were also monitored for adverse events (fever, vomiting, pain abdomen, or any other symptom).

### 2.2. Sample Size

This was calculated as per the method adopted by Teran et al. [13]. In order to detect an average difference of 24 hrs between the intervention and the control group, we assumed a standard deviation for the duration of diarrhea in the control group of 30 hrs. To achieve the power of 80% and a significance level of 5%, a total of 50 cases were needed (25 in each group).

### 2.3. Outcome Measures

*Primary*

1. Duration (in hrs) of acute diarrhea

*Secondary*

1. Duration (in hrs) of hospitalization
2. Duration (in hrs) of vomiting
3. Duration (in hrs) of fever
4. Proportion of children requiring parenteral rehydration
5. Any adverse effects

Duration of acute diarrhea was defined as the time (in hrs) from the first to the last abnormal (loose or liquid) stools preceding a normal stool output. Consistency of stool was evaluated through a score system, as described by previously [15], and stool was graded as 1 (normal), 2 (loose), 3 (semiliquid), and 4 (liquid). Duration of hospitalization was defined as the time from admission till discharge. Children were discharged 24 hrs after resolution of diarrhea. Adverse effects of nitazoxanide were also studied.

### 2.4. Statistical Analysis

All the data were entered into the Microsoft excel sheet. The data were analysed using SPSS software (version 20.0 Chicago, IL, USA). Statistical tests used for comparison included Chi-Square and the Mann–Whitney *U* test. Because many of the continuous variables were not normally distributed, when a Mann–Whitney *U* test was used to compare the groups, the medians and interquartile ranges (IQR) were presented. Difference between the median and calculation of 95% CI (confidence interval) was done [14]. Intention-to-treat analysis was used for the primary outcome. A *p* value of < 0.05 was considered significant.

## 3. Results

**Figure 1:**
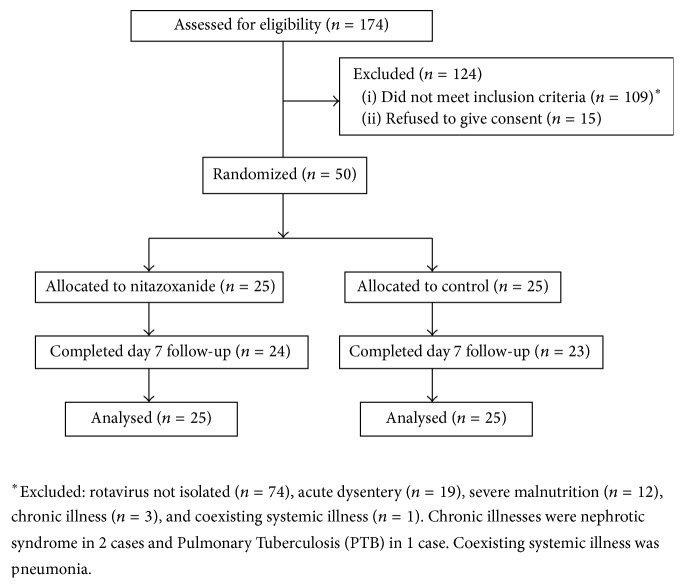
Flow of participants in the study.

Of 174 acute diarrhea cases, 109 were excluded for various reasons (details of the exclusion have been described in Figure 1). Of 65 eligible children, 50 finally enrolled in the study; 47 (94%) completed the day 7 follow-up (Figure 1). One child in the nitazoxanide group and 2 children in the control group did not come for day 7 follow-up. The clinical and demographic characteristics in the two groups were comparable. Demographic status and characteristics of acute diarrhea between the two groups were comparable (Table 1).

### 3.1. Primary Outcome Measures

*Duration of Acute Diarrhea*. In the nitazoxanide group, the median duration was significantly less by about 26 hrs [95% CI: –13.2 to –38.8] compared to the control group (Table 2).

### 3.2. Secondary Outcome Measures

*Duration of Hospitalization*. In the nitazoxanide group, the median duration was significantly less by about 22 hrs [95% CI: –12.98 to –31.02] compared to the control group (Table 2).

*Duration of Fever*. In the nitazoxanide group, the median duration was less by about 4 hrs [95% CI: –10.89 to 2.89] compared to the control group, but the result was nonsignificant (Table 2).

*Effect on Vomiting*. In the nitazoxanide group, the median duration was less by about 3 hrs [95% CI: –8.84 to 2.84] compared to the control group, but the result was nonsignificant (Table 2).

*Proportion of Children Requiring Parenteral Rehydration*. There was no significant difference between the two groups (odds ratio (OR) 0.48 [95% CI: 0.04 to 5.65]) (Table 2).

*Adverse Events*. There was no report of any adverse events in either of the groups.

## 4. Discussion

In this randomized controlled trial, nitazoxanide (100 mg in 12–47 months; 200 mg in ≥4 yrs) given orally twice daily for 3 days to children aged 12 months to 5 years during an acute episode of rotavirus diarrhea resulted in significant decrease in the duration of diarrhea and hospitalization without any adverse events. There was no effect on the duration of fever or vomiting or on the proportion of children requiring parenteral rehydration.

In the present study, the incidence of rotavirus diarrhea was 37.3%. Slightly lower or higher results (the range being 34% to 55%) have been obtained by others from India and outside [4, 16–19]. Now, there has been gradual introduction of rotavirus vaccine (monovalent, RV1, and pentavalent, RV5) in developing world, so the scenario might change in future. A Cochrane review found that in countries with high-mortality rates RV1 probably prevents 42% of severe rotavirus diarrhea cases, and RV5 prevents 41% of severe rotavirus diarrhea cases [20]. There has been a low but less variable efficacy of the vaccine in Indian setting. The efficacy is around 58% for Rotarix and 83% for Rotateq, by different ways of assessment [21, 22]. In 2014, another rotavirus vaccine (116E) showed 55% efficacy in a clinical trial in India [23]. Besides this, experience in other countries (Australian children) suggests that the vaccines are not targeting the proper strains of rotavirus, and some of the circulating serotypes are not responding to the vaccines or may be mutating. So, the vaccine alone may not be the answer for management of rotavirus diarrhea even after introduction of the vaccine.

Only two previous trials have been published till date on the role of nitazoxanide in treatment of acute rotavirus diarrhea in hospital settings from developed countries. In the first trial, 38 children (5 months to 7 years) were included and the intervention group received 7.5 mg/kg nitazoxanide orally twice daily for 3 days [11]. The median time to resolution of illness was 31 hrs for the nitazoxanide group compared with 75 hrs for the control group (*p* = 0.0137), and no significant adverse effect was reported. In the second trial, 75 children (28 days to 24 months) were included and the intervention group (*n* = 25) received oral nitazoxanide (15 mg/kg/day) twice daily for 3 days [13]. The median duration of hospitalization (nitazoxanide, 81 hrs, control, 108 hrs; *p* = 0.017) and diarrhea (nitazoxanide, 54 hrs, control, 79 hrs; *p* = 0.009) was significantly reduced in the nitazoxanide group. The only adverse event noted was greenish discoloration of body fluid that spontaneously disappeared in follow-up. The present study result was in accordance with these trials without report of any adverse event and provides evidence that nitazoxanide is helpful in hospitalized children with <10% dehydration.

The strength of the present study is that it studied the effect of nitazoxanide in Indian setting with high mortality due to rotavirus diarrhea. As previous trials have been conducted in western countries, their findings cannot be extrapolated on Indian children due to a higher breast feeding rate, poor hygienic condition, and a distinct gut colonization status. The age range included 12 months to 5 years which is important, as diarrhea is an important cause of under-five mortality in developing countries in this age group. The effect on the requirement of rehydration was evaluated which is important from public health point of view. Potential limitations include the following: no genotyping of rotavirus strain was done due to cost constraints. Measurement of the volume of stool output (g/kg) was also not done, which has been cited in different diarrhea research studies as an additional important outcome. Cost-effective analysis was not done.

## 5. Conclusion

Rotavirus as a single agent is the major cause of acute diarrhea (37.3%) in infants and under-five children. Oral nitazoxanide was found to be effective and safe in the management of acute rotavirus diarrhea in Indian children.

## Figures and Tables

**Table 1:** Baseline and demographic characteristics of the study population.

	Nitazoxanide group (*n* = 25)	Control group (*n* = 25)	*p* value
Age, months (IQR)	26 (8.7)	25 (8.3)	0.38
Males (%)	16 (64)	17 (68)	0.76
Weight (kg) (IQR)	11.3 (2.2)	11.1 (2.8)	0.28
Median duration (IQR) of diarrhea before treatment (hours)	42 (13)	45 (11)	0.35
Stool consistency at the time of enrolment (%)			
Liquid	11 (44)	10 (40)	0.24
Semiliquid	05 (20)	07 (28)	
Loose	09 (36)	08 (32)	
Number (%) of children vomiting	13 (52)	11 (44)	0.57
Median (IQR) duration of vomiting (hours)	28 (13)	30 (12)	0.4
Number (%) of children with fever	09 (36)	07 (28)	0.55
Median (IQR) duration of fever (hours)	30 (16)	33 (15)	0.47
Dehydration status (%)			
Mild	08 (32)	09 (36)	0.65
Moderate	13 (52)	11 (44)	

IQR: Interquartile range.

**Table 2:** Primary and secondary outcome measures.

	Nitazoxanide group (*n* = 25)	Control group (*n* = 25)	Difference [95% CI]
Median (IQR) hours of diarrhea^a^	54 (45–65)	80 (65–89)	–26 [–13.2 to –38.8]
Median (IQR) hours of hospitalization^a^	68 (57–75)	90 (77–93)	–22 [–12.98 to –31.02]
Median (IQR) duration of fever (hours)^a^	43 (35–48)	47 (42–52)	–4 [–10.89 to 2.89]
Median (IQR) duration of vomiting (hours)^a^	38 (33–41)	41 (35–46)	–3 [–8.84 to 2.84]
Proportion (%) of children requiring parenteral rehydration^b^	1 (4)	2 (8)	0.48 [0.04 to 5.65]

^a^The difference between the two medians and calculation of 95% CI (confidence interval) has been done by the method proposed by Bonett and Price [14].

^b^Data expressed in odds ratio (OR) and 95% CI.
